# Association of piperacillin/tazobactam MIC and mortality in a cohort of ceftriaxone-resistant *Escherichia coli* bloodstream infections treated with piperacillin/tazobactam and carbapenems: a multicentric propensity score-weighted observational cohort study

**DOI:** 10.1093/jac/dkad404

**Published:** 2024-01-03

**Authors:** Emanuele Rando, Federica Salvati, Flavio Sangiorgi, Francesca Catania, Elisa Leone, Alessandra Oliva, Francesco Di Gennaro, Barbara Fiori, Francesca Cancelli, Sara Figliomeni, Francesca Bobbio, Federica Sacco, Davide Fiore Bavaro, Lucia Diella, Alessandra Belati, Annalisa Saracino, Claudio Maria Mastroianni, Massimo Fantoni, Rita Murri

**Affiliations:** Dipartimento di Sicurezza e Bioetica—Sezione di Malattie Infettive, Università Cattolica del Sacro Cuore, Rome, Italy; Dipartimento di Sicurezza e Bioetica—Sezione di Malattie Infettive, Università Cattolica del Sacro Cuore, Rome, Italy; Dipartimento di Sicurezza e Bioetica—Sezione di Malattie Infettive, Università Cattolica del Sacro Cuore, Rome, Italy; Dipartimento di Sicurezza e Bioetica—Sezione di Malattie Infettive, Università Cattolica del Sacro Cuore, Rome, Italy; Dipartimento di Sicurezza e Bioetica—Sezione di Malattie Infettive, Università Cattolica del Sacro Cuore, Rome, Italy; Department of Public Health and Infectious Diseases, Sapienza University of Rome, Rome, Italy; Department of Precision and Regenerative Medicine and Ionian Area (DiMePRe-J), Unit of Infectious Diseases, University of Bari ‘A. Moro’, Polyclinic Hospital, Bari, Italy; Dipartimento di Scienze di Laboratorio e Infettivologiche, Fondazione Policlinico Universitario A Gemelli IRCCS, Rome, Italy; Dipartimento di Scienze Biotecnologiche di Base, Cliniche Intensivologiche e Perioperatorie, Università Cattolica del Sacro Cuore, Rome, Italy; Department of Public Health and Infectious Diseases, Sapienza University of Rome, Rome, Italy; Department of Public Health and Infectious Diseases, Sapienza University of Rome, Rome, Italy; Department of Public Health and Infectious Diseases, Sapienza University of Rome, Rome, Italy; Department of Public Health and Infectious Diseases, Sapienza University of Rome, Rome, Italy; Department of Precision and Regenerative Medicine and Ionian Area (DiMePRe-J), Unit of Infectious Diseases, University of Bari ‘A. Moro’, Polyclinic Hospital, Bari, Italy; Department of Precision and Regenerative Medicine and Ionian Area (DiMePRe-J), Unit of Infectious Diseases, University of Bari ‘A. Moro’, Polyclinic Hospital, Bari, Italy; Department of Precision and Regenerative Medicine and Ionian Area (DiMePRe-J), Unit of Infectious Diseases, University of Bari ‘A. Moro’, Polyclinic Hospital, Bari, Italy; Department of Precision and Regenerative Medicine and Ionian Area (DiMePRe-J), Unit of Infectious Diseases, University of Bari ‘A. Moro’, Polyclinic Hospital, Bari, Italy; Department of Public Health and Infectious Diseases, Sapienza University of Rome, Rome, Italy; Dipartimento di Sicurezza e Bioetica—Sezione di Malattie Infettive, Università Cattolica del Sacro Cuore, Rome, Italy; Dipartimento di Scienze di Laboratorio e Infettivologiche, Fondazione Policlinico Universitario A Gemelli IRCCS, Rome, Italy; Dipartimento di Sicurezza e Bioetica—Sezione di Malattie Infettive, Università Cattolica del Sacro Cuore, Rome, Italy; Dipartimento di Scienze di Laboratorio e Infettivologiche, Fondazione Policlinico Universitario A Gemelli IRCCS, Rome, Italy

## Abstract

**Objectives:**

To assess the impact of piperacillin/tazobactam MICs on in-hospital 30 day mortality in patients with third-generation cephalosporin-resistant *Escherichia coli* bloodstream infection treated with piperacillin/tazobactam, compared with those treated with carbapenems.

**Methods:**

A multicentre retrospective cohort study was conducted in three large academic hospitals in Italy between 2018 and 2022. The study population comprised patients with monomicrobial third-generation cephalosporin-resistant *E. coli* bloodstream infection, who received either piperacillin/tazobactam or carbapenem therapy within 48 h of blood culture collection. The primary outcome was in-hospital 30 day all-cause mortality. A propensity score was used to estimate the likelihood of receiving empirical piperacillin/tazobactam treatment. Cox regression models were performed to ascertain risk factors independently associated with in-hospital 30 day mortality.

**Results:**

Of the 412 consecutive patients included in the study, 51% received empirical therapy with piperacillin/tazobactam, while 49% received carbapenem therapy. In the propensity-adjusted multiple Cox model, the Pitt bacteraemia score [HR 1.38 (95% CI, 0.85–2.16)] and piperacillin/tazobactam MICs of 8 mg/L [HR 2.35 (95% CI, 1.35–3.95)] and ≥16 mg/L [HR 3.69 (95% CI, 1.86–6.91)] were significantly associated with increased in-hospital 30 day mortality, while the empirical use of piperacillin/tazobactam was not found to predict in-hospital 30 day mortality [HR 1.38 (95% CI, 0.85–2.16)].

**Conclusions:**

Piperacillin/tazobactam use might not be associated with increased mortality in treating third-generation cephalosporin-resistant *E. coli* bloodstream infections when the MIC is <8 mg/L.

## Introduction

*Escherichia coli* represents one of the most frequent causes of bloodstream infection (BSI) and urinary tract infection (UTI) worldwide.^1^ In recent years, an increase in the incidence of infections caused by third-generation cephalosporin-resistant (3GCR) *E. coli* was observed across all countries.^2,3^ These strains represent a major concern in both hospital and community settings,^4,5^ accounting for increased mortality rates compared with susceptible ones.^6,7^

The best therapeutic strategy for managing these infections has long been debated.^8–11^ The MERINO-1 trial showed that piperacillin/tazobactam did not result in non-inferior 30 day mortality when compared with meropenem in patients with BSI caused by ceftriaxone-resistant *E. coli* or *K. pneumoniae*, suggesting the avoidance of piperacillin/tazobactam in this patient population.^12^ Due to this trial’s results, carbapenems are currently considered the therapy of choice in treating BSI caused by 3GCR *E. coli*. However, the role of β-lactam/β-lactamase inhibitors (BL/BLIs) still needs to be completely elucidated in specific circumstances,^13,14^ particularly regarding the possible impact of piperacillin/tazobactam MICs on therapy effectiveness in patients with 3GCR Enterobacterales infections. For instance, a MERINO-1 *post hoc* analysis confirmed the mortality trend observed in the two groups, although the differences were less pronounced in the piperacillin/tazobactam group after excluding piperacillin/tazobactam-non-susceptible strains. Moreover, piperacillin/tazobactam-non-susceptible isolates (MIC > 16 mg/L) were found to be predictors of 30 day mortality.^15^ Additionally, a recent study depicted better outcomes when piperacillin/tazobactam was prescribed for Enterobacterales infections in the presence of an MIC of ≤16/4 mg/L compared with ≥32/4 mg/L.^16^

Either way, recent years have seen an increase in meropenem prescriptions to treat 3GCR *E. coli* BSI, potentially contributing to the global spread of carbapenemase-producing Enterobacterales.^17,18^ In this setting, understanding potential situations where piperacillin/tazobactam might still be valuable would provide alternatives to carbapenems to reduce selection pressure and control the increase in carbapenem-resistant pathogens. For these reasons, we hypothesized that piperacillin/tazobactam MICs could predict mortality outcomes in patients with BSI caused by 3GCR *E. coli,* offering a piece of real-world new evidence following the findings of the *post hoc* analysis of the MERINO-1 trial. Therefore, in this study, we aimed to assess the impact of piperacillin/tazobactam MICs on in-hospital 30 day mortality in a cohort of patients with 3GCR *E. coli* monomicrobial BSI treated with piperacillin/tazobactam compared with patients treated with carbapenems.

## Methods

### Study design and setting

We performed a multicentre cohort study of patients with 3GCR *E. coli* BSI diagnosed in three large academic hospitals in Italy, specifically Fondazione Policlinico Universitario Agostino Gemelli IRCCS in Rome, Policlinico Umberto I in Rome and the Ospedale Policlinico di Bari, from January 2018 to March 2022. Patients were identified from single centres’ electronic microbiology databases or administrative records. Patient information was anonymized and de-identified prior to data collection and analysis. Follow-up was conducted by analysing patients’ electronic in-hospital medical records until 30 days after the date of blood culture collection.

The study was performed following the Declaration of Helsinki and was approved by the Ethics Committee of the Fondazione Policlinico Universitario Agostino Gemelli IRCCS, Policlinico Umberto I, and Ospedale Policlinico di Bari (reference number ID 4992). In accordance with the committee recommendations, written informed consent or proxy consent was waived due to the study’s retrospective observational design.

### Participants

Patients meeting all the following criteria were included: (i) monomicrobial 3GCR *E. coli* BSI; (ii) receiving empirical therapy with piperacillin/tazobactam or carbapenems within 48 h of blood culture collection regardless of patient’s prognosis, and (iii) entire therapy course with piperacillin/tazobactam or carbapenems. Patients with a polymicrobial BSI and patients on combination antibiotic therapy were excluded. If a patient experienced two or more episodes of BSI, only the first event was included.

### Exposure and outcome

Empirical therapy with piperacillin/tazobactam or carbapenems was defined as a monoantimicrobial use before susceptibility was known and started within 48 h of blood culture collection. Definitive therapy with piperacillin/tazobactam or carbapenems was defined as a monodrug intake for ≥50% of the total duration of antibiotic therapy after *in vitro* susceptibility was known, regardless of which antibiotic was started first.

Based on the above defined exposures, patients were divided into two different populations, the ITT-like population made up of patients receiving empirical therapy with piperacillin/tazobactam or carbapenems, and the as-treated (AT) population receiving ≥50% of therapy with piperacillin/tazobactam or carbapenems.

The primary outcome was in-hospital 30 day all-cause mortality from blood culture collection in the ITT-like and AT populations. In-hospital 14 day all-cause mortality and length of stay were the secondary outcomes. The latter was defined as the number of days from blood culture collection to death or discharge.

### Variables and definition

Data collected included patient demographics, pre-existing medical conditions, Charlson’s co-morbidity index (CCI), severe immunocompromised status (defined as a solid organ or stem cell transplant, HIV infection with a CD4 count of <200/mm^3^, chemotherapy within 6 months, or receipt within 30 days of prednisone ≥10 mg/day or equivalent corticosteroid dose, or tumour necrosis factor α inhibitor or other directed monoclonal immunomodulatory antibody), the severity of illness at the time of blood culture collection (Pitt bacteraemia score), likely source of BSI, BSI setting (clinical or surgical ward), inflammatory laboratory markers, length of stay and detailed antibiotic administration including dosage, specific carbapenem used and duration of antibiotic therapy.

Microbiological data, including *E. coli* piperacillin/tazobactam and carbapenem MICs, as well as any resistance genes, were identified according to each laboratory method.

### Microbiology

At Fondazione Policlinico Universitario Agostino Gemelli IRCCS, the aliquots from each positive blood culture bottle were subjected to routine Gram-staining microscopy and solid-medium subcultures. After isolation from the cultures, bacteria and yeasts were identified by a MALDI-TOF MS system. Antimicrobial susceptibility testing of the bacterial isolates was performed with the VITEK 2 (bioMérieux) and/or ETEST (bioMérieux) and interpreted according to EUCAST guidelines. At Policlinico Umberto I, the positive blood cultures for Gram-negative bacilli were cultured on agar media and incubated for 24 h at 37°C. Isolated strains were identified using a MALDI-TOF MS system (Bruker Daltonik, Bremen, Germany) and antimicrobial susceptibility testing was performed by MicroScan Walkaway (Beckman and Coulter, Brea, CA, USA) system. Regarding Ospedale Policlinico di Bari, samples were collected for the microbiology assessment before starting empirical antimicrobial therapy. According to current guidelines, blood cultures were performed by collecting 15–20 mL of blood per culture set. Two bottles per set were used and immediately placed into a BacT/ALERT 3D instrument (bioMérieux Inc., Marcy-l’Étoile, France). Positive aerobic blood cultures were subcultured on MacConkey agar, CNA blood agar, Sabouraud dextrose agar, mannitol-salt agar and chocolate agar, and incubated aerobically at 37°C for 24 h. Identification and antibacterial susceptibility were tested on the automated VITEK 2 system and VITEK MS (bioMérieux) according to the manufacturer’s instructions. The interpretative breakpoints for MIC values were based on the EUCAST criteria.

To identify the resistance mechanism, at Fondazione Policlinico Universitario Agostino Gemelli IRCCS, the NG-Test^®^ CTX-M MULTI was used. This test is a qualitative lateral flow immunoassay for the rapid detection of the five major groups in the CTX-M-type enzymes of ESBLs produced by Enterobacterales. Rapid tests detect enzymes belonging to CTX-M Groups 1, 2, 8, 9 and 25, including their most clinically relevant variants in the same cassette, in less than 15 min. This test was available only at the abovementioned institution.

### Statistical analysis

Continuous variables were described using medians and IQRs, and categorical variables using frequencies and percentages. Wilcoxon rank-sum test was used to compare continuous variables and Fisher’s exact test for categorical variables. A *P* value of <0.05 was used to consider differences as statistically significant. Multiple imputations by chained equation (MICE) with five cycles were performed for variables with <5% of missing data, excluding outcome data. Since the group comparisons were potentially affected by small sample sizes, standardized differences (SD) were calculated by dividing the difference between the groups by the pooled standard deviation of the two groups. SD > 0.1 was interpreted as a meaningful difference.

A propensity score (PS) of receiving empirical piperacillin/tazobactam was estimated using a generalized boosted model due to the variety of response variables and the absence of formal distributional assumptions. Covariates to include in the PS were identified by selecting variables with SD > 0.1 in the comparison between piperacillin/tazobactam versus carbapenem empirical therapy and with SD > 0.1 between survivors versus non-survivors. A patient who was treated with empirical piperacillin/tazobactam was weighted by the inverse of the probability that they would be treated with empirical piperacillin/tazobactam, and a patient who did not receive empirical piperacillin/tazobactam was weighted by the inverse of the probability that they would not receive empirical piperacillin/tazobactam, equivalent to 1 minus their propensity score. After that, crude and propensity-weighted simple and multiple Cox regression models were performed to ascertain risk factors independently associated with in-hospital 30 day mortality. A Cox regression strategy was preferred due to the unavailable 30 day follow-up regarding the outcome status since patients were censored at discharge. Variables in the model were included if they had an influence on the in-hospital 30 day mortality outcome based on clinical meaningfulness by investigator consensus and had SD > 0.25 in the weighted comparison between the exposure groups. HR and 95% CI were calculated. Cox proportional hazards assumptions of the models were verified. After that, we performed 1000 bootstrap resamples of the data to estimate robust HR for the simple and multiple PS-adjusted Cox regression, given the potential alterations induced by the pseudopopulation created by the PS-weighting analysis.

Survival analysis was performed using both the crude and propensity-adjusted Kaplan–Meier curves. A non-parametric (log-rank) test was used to define their statistical significance.

Statistical analyses were retrospectively performed with R software version 4.2.2 and RStudio 2023.03.0 + 386 [R Core Team (2020). R: A language and environment for statistical computing. R Foundation for Statistical Computing, Vienna, Austria. https://www.R-project.org/].

## Results

### Group characteristics

**Figure 1.**
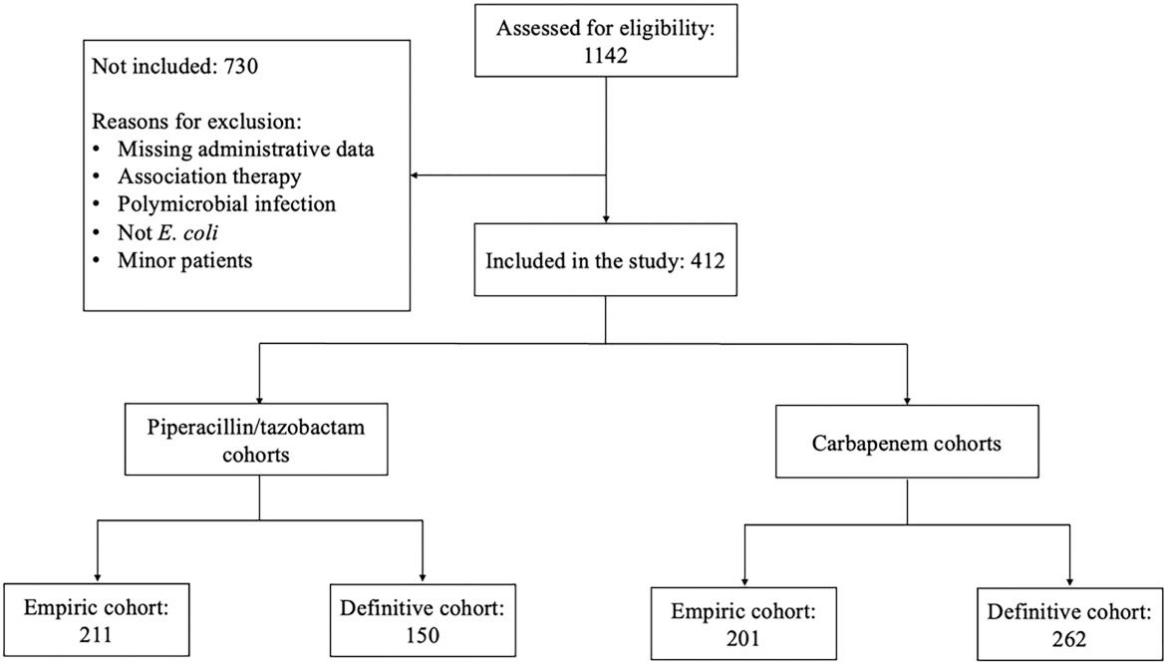
Study diagram.

Overall, 1142 patients were assessed for eligibility, and 730 of them were excluded due to reasons reported in Figure 1. Finally, 412 were included, according to the inclusion criteria, and subsequently analysed.

The main characteristics of the general population and patients who received empirical and definitive therapy are summarized in Table 1. Two hundred and eleven patients (51%) received empirical therapy with piperacillin/tazobactam, while 201 (49%) received a carbapenem. Immunocompromised patients [64 (32%) versus 39 (18%), *P* = 0.002], those with antibiotic therapy in the previous 30 days [66 (38%) versus 50 (27%), *P* = 0.032], and those with MDR isolates in the previous 90 days [38 (22%) versus 18 (10%), *P* = 0.002), were more likely to receive carbapenem empirical treatment.

**Table 1.** Characteristics of the empirical and definitive cohorts

	Empirical therapy			Definitive therapy		
Characteristic	Piperacillin/tazobactam, *N* = 211^a^	Carbapenem, *N* = 201^a^	Significance^b^	Piperacillin/tazobactam, *N* = 150^a^	Carbapenem, *N* = 262^a^	Significance^b^
Age	74 (63–83)	76 (67–82)		74 (63–82)	76 (65–82)	
Female sex	103/211 (49)	88/201 (44)		79/150 (53)	112/262 (43)	
Infection acquisition						
* *Community-acquired	102/211 (48)	94/201 (47)		72/150 (48)	124/262 (47)	
* *Hospital-acquired	84/211 (40)	81/201 (40)		61/150 (41)	104/262 (40)	
* *Healthcare-associated	25/211 (12)	26/201 (13)		17/150 (11)	34/262 (13)	
Source						
* *UTI	105/211 (50)	107/201 (53)		79/150 (53)	133/262 (51)	
* *Surgical site infection	4/211 (1.9)	3/201 (1.5)		2/150 (1.3)	5/262 (1.9)	
* *Pneumonia	3/211 (1.4)	4/201 (2.0)		2/150 (1.3)	5/262 (1.9)	
* *CVC-related	3/211 (1.4)	2/201 (1.0)		2/150 (1.3)	3/262 (1.1)	
* *cIAI	23/211 (11)	11/201 (5.5)		15/150 (10)	19/262 (7.3)	
* *Mucositis	3/211 (1.4)	1/201 (0.5)		1/150 (0.7)	3/262 (1.1)	
* *ABSSTI	1/211 (0.5)	1/201 (0.5)		0/150 (0)	2/262 (0.8)	
* *Biliary tract infection	23/211 (11)	19/201 (9.5)		17/150 (11)	25/262 (9.5)	
* *Other	4/211 (1.9)	4/201 (2.0)		1/150 (0.7)	7/262 (2.7)	
* *Unknown	42/211 (20)	49/201 (24)		31/150 (21)	60/262 (23)	
* *Urinary/biliary tract infection	128/211 (61)	126/201 (63)		96/150 (64)	158/262 (60)	
* *Immunocompromised	39/211 (18)	64/201 (32)	**	36/150 (24)	67/262 (26)	
* *CVC	46/199 (23)	65/189 (34)	*	30/140 (21)	81/248 (33)	*
Long-term care in prior 90 days	92/197 (47)	111/186 (60)	*	66/142 (46)	137/241 (57)	
MDR isolation in prior 90 days	18/184 (9.8)	38/171 (22)	**	12/133 (9.0)	44/222 (20)	**
Antibiotic use in prior 30 days	50/183 (27)	66/173 (38)	*	35/135 (26)	81/221 (37)	*
Moderate to severe chronic kidney disease	36/211 (17)	45/201 (22)		24/150 (16)	57/262 (22)	
C-reactive protein (mg/L)	141 (48–213)	137 (38–195)		156 (61–224)	128 (39–192)	*
Procalcitonin (ng/mL)	4 (1–17)	6 (1–20)		4 (1–17)	5 (1–21)	*
CTX-M positive	115/211 (55)	112/201 (56)		80/150 (53)	147/262 (56)	
TZP MIC (mg/L)						
≤4	144/211 (68)	116/201 (58)		121/150 (81)	139/262 (53)	
8	44/211 (21)	52/201 (26)		22/150 (15)	74/262 (28)	
16	6/211 (2.8)	8/201 (4.0)		1/150 (0.7)	13/262 (5.0)	
≥32	17/211 (8.1)	25/201 (12)		6/150 (4.0)	36/262 (14)	
Meropenem MIC (mg/L)						
≤0.25	210/211 (100)	199/201 (99)		149/150 (99)	260/262 (99)	
1	1/211 (0.5)	0/201 (0)		1/150 (0.7)	0/262 (0)	
8	0/211 (0)	2/201 (1.0)		0/150 (0)	2/262 (0.8)	
Carbapenem definitive therapy	81/211 (38)	181/201 (90)	***	20/150 (13)	181/262 (69)	***
CCI	6.00 (5.00–8.00)	7.00 (5.00–9.00)		6.00 (4.00–8.00)	7.00 (5.00–8.00)	
Pitt bacteraemia score	0 (0–1)	0 (0–2)		0 (0–1)	0 (0–2)	
In-hospital 14 day mortality	24/211 (11)	18/201 (9.0)		14/150 (9.3)	28/262 (11)	
In-hospital 30 day mortality	29/211 (14)	25/201 (12)		17/150 (11)	37/262 (14)	

TZP, piperacillin/tazobactam; ABSSTI, acute bacterial skin and skin structure infection. *P ≤ 0.05, **P ≤ 0.01, ***P ≤ 0.001.

^a^Median (IQR) or frequency (%).

^b^Wilcoxon rank-sum test; Fisher’s exact test.

The overall in-hospital 14 day mortality was 10% (*n* = 42), and the in-hospital 30 day mortality was 13% (*n* = 54). Eight patients died within 48 h from blood culture collection, five in the empirical piperacillin/tazobactam group and three in the empirical carbapenem group. There were no differences in mortality between piperacillin/tazobactam versus carbapenem empirical therapy and piperacillin/tazobactam versus carbapenem definitive therapy (Table 1).

Piperacillin/tazobactam dosage data for the definitive cohort were available for 116 patients; 92 (79%) were on the 4.5 g every 8 h dosage. Regarding carbapenem prescriptions, 248 (91%) were on meropenem 1 g every 8 h; the rest were on ertapenem 1 g every 24 h (*n* = 14; 5%) or imipenem 1 g every 8 h (*n* = 11; 4%).

Almost all *E. coli* strains (*n* = 409; 99%) had a meropenem MIC of ≤0.25 mg/L, while 152 of the strains (37%) had a piperacillin/tazobactam MIC of >4 mg/L. Table 2 reports the main characteristics of patients with BSI caused by *E. coli* strains with piperacillin/tazobactam MIC ≤ 4 mg/L versus >4 mg/L. SD differences between the two empirical treatment groups and survivors versus non-survivors are reported in Table S1 (available as Supplementary data at *JAC* Online).

**Table 2.** Piperacillin/tazobactam MIC ≤ 4 mg/L versus MIC > 4 mg/L characteristics

		Piperacillin/tazobactam MIC		
Characteristic	Overall, *N* = 412^a^	≤ 4 mg/L, *N* = 260^a^	> 4 mg/L, *N* = 152^a^	Significance^b^
Age	75 (64–82)	75 (64–81)	76 (64–83)	
Female sex	191/412 (46)	129/260 (50)	62/152 (41)	
Infection acquisition				
Community-acquired	196/412 (48)	127/260 (49)	69/152 (45)	
Hospital-acquired	165/412 (40)	107/260 (41)	58/152 (38)	
Healthcare-associated	51/412 (12)	26/260 (10)	25/152 (16)	
Source				
UTI	212/412 (51)	137/260 (53)	75/152 (49)	
Surgical site infection	7/412 (1.7)	3/260 (1.2)	4/152 (2.6)	
Pneumonia	7/412 (1.7)	4/260 (1.5)	3/152 (2.0)	
CVC-related	5/412 (1.2)	3/260 (1.2)	2/152 (1.3)	
cIAI	34/412 (8.3)	22/260 (8.5)	12/152 (7.9)	
Mucositis	4/412 (1.0)	2/260 (0.8)	2/152 (1.3)	
ABSSTI	2/412 (0.5)	0/260 (0)	2/152 (1.3)	
Biliary tract infection	42/412 (10)	26/260 (10)	16/152 (11)	
Other	8/412 (1.9)	4/260 (1.5)	4/152 (2.6)	
Unknown	91/412 (22)	59/260 (23)	32/152 (21)	
Urinary/biliary tract infection	254/412 (62)	163/260 (63)	91/152 (60)	
Immunocompromised	103/412 (25)	67/260 (26)	36/152 (24)	
CVC	111/388 (29)	64/243 (26)	47/145 (32)	
Long-term care in prior 90 days	203/383 (53)	117/242 (48)	86/141 (61)	*
MDR isolation in prior 90 days	56/355 (16)	31/224 (14)	25/131 (19)	
Antibiotic use in prior 30 days	116/356 (33)	65/226 (29)	51/130 (39)	*
Moderate to severe chronic kidney disease	81/412 (20)	44/260 (17)	37/152 (24)	
CTX-M positive	227/412 (55)	146/260 (56)	81/152 (53)	
CCI	7.00 (5.00–8.00)	6.00 (5.00–8.00)	7.00 (5.00–8.00)	
Pitt bacteraemia score	0 (0–1)	0 (0–1)	0 (0–1)	
In-hospital 14 day mortality	42/412 (10)	16/260 (6.2)	26/152 (17)	***
In-hospital 30 day mortality	54/412 (13)	22/260 (8.5)	32/152 (21)	***

ABSSTI, acute bacterial skin and skin structure infection. *P ≤ 0.05, **P ≤ 0.01, ***P ≤ 0.001.

^a^Median (IQR) or frequency (%).

^b^Wilcoxon rank-sum test; Fisher’s exact test.

### Cox regression and survival analysis

A PS was calculated. Variables with SD > 0.1 for both the exposure and outcome group included in the PS were: age, medical ward stay, surgical ward stay, complicated intrabdominal infection (cIAI), BSI source, unknown BSI source, coronary artery disease, heart failure, dementia, leukaemia/lymphoma, COPD, liver disease, chronic kidney disease, AIDS and CCI. The balance of the propensity model was evaluated by verifying the obtained balance of PS covariates (Figure S1).

Cox regression models for in-hospital 30 day mortality and survival analysis were performed. Variables included in both crude and PS-adjusted models were empirical piperacillin/tazobactam, immunocompromised status, CCI, Pitt bacteraemia score and piperacillin/tazobactam MIC of 8 mg/L and ≥16 mg/L (Figure S1).

In the crude multiple Cox regression model, the Pitt bacteraemia score [HR 1.23 (95% CI, 1.10–1.38)], MIC of piperacillin/tazobactam of 8 mg/L [HR 2.29 (95% CI, 1.25–4.21)] and MIC ≥ 16 mg/L [HR 3.04 (95% CI, 1.49–6.19)] were significantly associated with in-hospital 30 day mortality.

The propensity-adjusted simple Cox regression model yielded an HR for empirical piperacillin/tazobactam of 1.28 (95% CI, 0.83–1.99). In the propensity-adjusted multiple Cox model, empirical piperacillin/tazobactam [HR 1.38 (95% CI, 0.85–2.16)] was not found to predict in-hospital 30 day mortality. In comparison, the Pitt bacteraemia score [HR 1.26 (95% CI, 1.13–1.40)], piperacillin/tazobactam MIC of 8 mg/L [HR 2.35 (95% CI, 1.35–3.95)] and piperacillin/tazobactam MIC ≥ 16 mg/L [HR 3.69 (95% CI, 1.86–6.91)] were found to be significantly associated with in-hospital 30 day mortality. Complete HRs and 95% CIs of both models are reported in Table 3.

**Table 3.** Multiple Cox regression models for in-hospital 30 day mortality

	Crude model		Propensity-adjusted	
Characteristic	HR	95% CI	HR	95% CI
Empirical TZP	1.34	0.78–2.32	1.38	0.85–2.16
Immunocompromised	0.88	0.47–1.66	0.79	0.43–1.41
CCI	1.07	0.96–1.19	1.08	0.98–1.17
Pitt bacteraemia score	1.23	1.10–1.38	1.26	1.13–1.40
TZP MIC (mg/L)				
8	2.29	1.25–4.21	2.35	1.35–3.95
≥16	3.04	1.49–6.19	3.69	1.86–6.91

CI, confidence interval; HR, hazard ratio; TZP, piperacillin/tazobactam.

Cox proportional hazards assumptions were verified (Figure S2).

**Figure 2.**
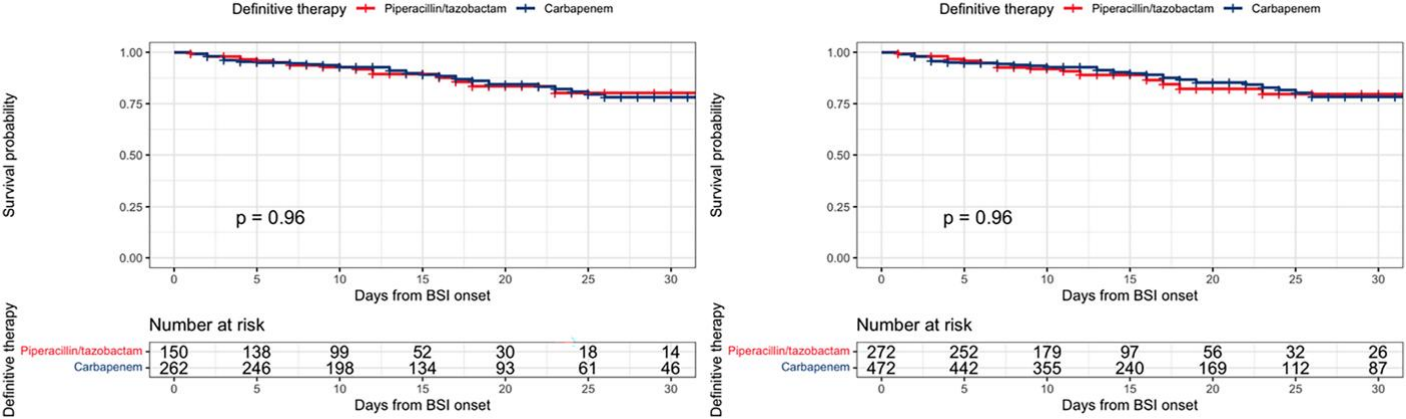
Crude and propensity-adjusted Kaplan–Meier curves of definitive therapy cohorts. This figure appears in colour in the online version of *JAC* and in black and white in the print version of *JAC*.

The crude and propensity-adjusted Kaplan–Meier curves for definitive piperacillin/tazobactam or carbapenems therapy are reported in Figure 2. The log-rank test was *P* = 0.96.

## Discussion

In our retrospective observational study, the use of piperacillin/tazobactam in both empirical and definitive therapy did not result in increased in-hospital 30 day mortality compared with carbapenems. Although empirical piperacillin/tazobactam was not predictive of in-hospital 30 day mortality, we found that piperacillin/tazobactam MIC at the breakpoint level of 8 mg/L and ≥16 mg/L was significantly associated with in-hospital 30 day mortality both in the crude and propensity-weighted multiple Cox regression. Conflicting evidence exists regarding the impact of piperacillin/tazobactam MIC on mortality for BSI caused by Enterobacterales, among which is *E. coli.* For instance, a previous observational study showed no trend towards increased clinical failure or mortality in patients with BSI caused by ESBL-producing Enterobacterales when MICs were borderline (between 8 and 16 mg/L) compared with MICs of ≤4 mg/L.^19^ In contrast, after reanalysing in-hospital 30 day mortality considering only piperacillin/tazobactam-sensitive strains of the MERINO-1 trial, the difference between piperacillin/tazobactam and carbapenem groups was attenuated.^15^ Moreover, it was found that the piperacillin/tazobactam-non-susceptible breakpoint (MIC > 16 mg/L) was a predictor of in-hospital 30 day mortality after accounting for confounders.^15^ However, it must be noted that the trial was not powered to detect potential differences in subgroups. Finally, more recent evidence reported better outcomes when the MIC was <16/4 mg/L compared with ≥32/4 mg/L in patients with Enterobacterales infections receiving piperacillin/tazobactam therapy.^16^ In our study, piperacillin/tazobactam susceptibility was tested by standardized methods for all 3GCR *E. coli* strains, considering strains as resistant even in the presence of the EUCAST breakpoint of 8 mg/L, without excluding other isolates from the analysis. Interestingly, both 8 and ≥16 mg/L piperacillin/tazobactam MICs were significant predictors of in-hospital 30 day mortality. Overall, our results highlight that attention must be paid to the pathogen’s antibiotic resistance profile, even when the MIC is at the breakpoint level, in order not to administer antibiotic therapy that may impact survival, as previously reported in the literature.^20,21^ This fact can also be explained by the intrinsic variation in MIC determination, especially for high MICs.^22^ In addition, we found lower in-hospital 14 day mortality in patients with urinary/biliary-source BSI, while patients with an intra-abdominal source were less likely to survive. The urinary and biliary sources are known to be associated with lower mortality rates compared with other sources, and higher mortality has been described in abdominal-onset BSI.^23,24^ This finding may be because piperacillin/tazobactam and carbapenems concentrate well in the urinary tract, and source control is generally more readily performed. In contrast, source control is often delayed or not performed completely in intra-abdominal infections. Besides, the inoculum effect seems to affect piperacillin/tazobactam more than carbapenems, complicating the treatment of cIAI compared with urinary/biliary infections.^25^

Some limitations of this study must be considered. Firstly, concerns about unmeasured confounders are inherently part of the study’s observational design, and since randomization was not possible, the two cohorts presented imbalances in several characteristics. For instance, immunocompromised patients, patients with previous MDR isolates and those previously receiving antibiotic therapy were more frequently given empirical antibiotic therapy with carbapenems. This imbalance may highlight the clinician’s propensity to empirically administer carbapenems in certain patients potentially with a higher risk of dying because of their baseline conditions. In addition, the sample size could not guarantee sufficient power considering the observational design and the use of the Cox regression.

Secondly, the exact dosage of the administered drug was available only for a few patients and drug dilution schemes and infusion rates (i.e. bolus versus intermittent versus continuous infusion) were not standardized in every medical department, so the possible influence of differences in drug blood concentration on the results cannot be excluded. For these reasons, variability in antibiotic infusion times was likely. This fact may limit the study’s interpretation. For instance, current EUCAST guidelines do not recommend piperacillin/tazobactam dosage less than every 6 h in bolus or every 8 h over a 4 hour infusion in infections caused by 3GCR isolates. Thirdly, only *E. coli* isolates were included. This fact reduces the study’s generalizability, especially regarding *K. pneumoniae* and other Enterobacterales. This choice was due to the high local piperacillin/tazobactam resistance rates of *K. pneumoniae*, for which piperacillin/tazobactam is less used as empirical therapy, restricting the available sample size for this pathogen. Nevertheless, the inclusion of only *E. coli* infections may also be an added value of this study, specifically offering a pathogen-based view when treating this organism. Fourthly, CTX-M expression is the only 3GCR mechanism routinely screened in our laboratories. Because of this, tracing the co-production of additional penicillinases potentially influencing piperacillin/tazobactam resistance was not feasible. Indeed, not knowing the exact resistance mechanism limits the study appraisal, and its potential consequences on the specific drugs used in the study. In this regard, other penicillinases not susceptible to tazobactam action have been described in some studies.^26^ The OXA-1 penicillinase, for instance, presents low susceptibility to tazobactam action. A study showed that *bla*_OXA-1_ expression in patients with ESBL *E. coli* BSI was associated with significantly increased piperacillin/tazobactam MIC up to 8 or 16 mg/L.^27^ Interestingly, OXA genes were found in 102 strains isolated from MERINO-1 participants, with OXA-1 as the main gene detected.^15^ Therefore, since OXA-1 has not been routinely tested, co-expression of other potential resistance pathways associated with increased piperacillin/tazobactam MIC could not be ruled out. Moreover, sensitivity to amoxicillin/clavulanate, as a surrogate for the possible presence of OXA-1, could not be reported. A further limitation of our study is the heterogeneity of measurements. Antibiotic susceptibility tests were performed on samples from hospitals with different laboratory methods. Phenotypic methods for MIC measurement were used, specifically Hospital Umberto I used MicroScan, Hospital Gemelli used VITEK-2 and/or ETEST and Bari used VITEK 2. While almost all isolates had meropenem MIC value within the WT distribution, piperacillin/tazobactam MIC distribution was very close to the clinical breakpoint (≥8 mg/L according to the current EUCAST susceptibility breakpoint), for which the possibility of misclassification into ‘susceptible’ or ‘resistant’ due to imprecision of the method was possible.

Fifthly, since most data were collected from administrative records, details on source control were not collected. For this reason, it was not possible to adjust for this vital confounder. However, according to each intrahospital and good practice policy, source control procedures were carried out as soon as possible.

In conclusion, our study highlighted that piperacillin/tazobactam use might not be associated with increased mortality in treating 3GCR *E. coli* BSI when the MIC is <8 mg/L. In low-prevalence piperacillin/tazobactam resistance settings, starting empirical piperacillin/tazobactam therapy in the presence of ESBL-producing *E. coli* BSI might be considered, especially for lower inoculum effect infections. Additionally, if piperacillin/tazobactam was initiated as empirical therapy and clinical improvement occurs, no change of antibiotic therapy could be necessary, thus expanding the indications of 2023 IDSA Guidance on the Treatment of Antimicrobial Resistant Gram-Negative Infections. Finally, according to the purpose of applying a proper carbapenem-sparing strategy, de-escalation to piperacillin/tazobactam might be reasonable once the definitive antimicrobial susceptibility test results are available. Considering the increasing carbapenem resistance, further studies assessing the dosages and administration regimens of piperacillin/tazobactam are encouraged.

## Supplementary Material

dkad404_Supplementary_Data

## Data Availability

The datasets used and/or analysed during the current study are available from the corresponding author upon reasonable request.

## Supplementary data

Figures S1 and S2 and Table S1 are available as Supplementary data at *JAC* Online.
