# Supplementary material for: Association of piperacillin/tazobactam MIC and mortality in a cohort of ceftriaxone-resistant Escherichia coli bloodstream infections treated with piperacillin/tazobactam and carbapenems: a multicentric propensity score-weighted observational cohort study
Source: J Antimicrob Chemother. 2024 Jan 3;79(2):453–61. doi: 10.1093/jac/dkad404 (PMC10832597; doi:10.1093/jac/dkad404)
Supplement: dkad404_Supplementary_Data [file dkad404_supplementary_data.docx]

**Table S1. Standardised mean differences of the empiric treatment cohorts and mortality cohorts**

|  | **Empiric treatment** | | | **Mortality** | | |
| --- | --- | --- | --- | --- | --- | --- |
| **Characteristic** | **Piperacillin/tazobactam**, N = 211^1^ | **Carbapenem**, N = 201^1^ | **SMD**^2^ | **Survivors**, N = 358^1^ | **Non-survivors**, N = 54^1^ | **SMD**^2^ |
| **Age** | 70.98 (15.67) | 72.57 (13.42) | 0.109 | 71.00 (14.88) | 76.74 (11.71) | 0.429 |
| **Female sex** | 103 (48.8) | 88 (43.8) | 0.101 | 166 (46.4) | 25 (46.3) | 0.001 |
| **Medical ward** | 150 (71.1) | 157 (78.1) | 0.162 | 264 (73.7) | 43 (79.6) | 0.140 |
| **Surgical ward** | 53 (25.1) | 36 (17.9) | 0.176 | 82 (22.9) | 7 (13.0) | 0.261 |
| **ICU** | 8 (3.8) | 8 (4.0) | 0.010 | 12 (3.4) | 4 (7.4) | 0.180 |
| **Community-acquired** | 102 (48.3) | 94 (46.8) | 0.032 | 169 (47.2) | 27 (50.0) | 0.056 |
| **Hospital-acquired** | 84 (39.8) | 81 (40.3) | 0.010 | 146 (40.8) | 19 (35.2) | 0.116 |
| **Healthcare-associated** | 25 (11.8) | 26 (12.9) | 0.033 | 43 (12.0) | 8 (14.8) | 0.082 |
| **UTI** | 105 (49.8) | 107 (53.2) | 0.069 | 191 (53.4) | 21 (38.9) | 0.293 |
| **SSI** | 4 (1.9) | 3 (1.5) | 0.031 | 6 (1.7) | 1 (1.9) | 0.013 |
| **Pneumonia** | 3 (1.4) | 4 (2.0) | 0.044 | 5 (1.4) | 2 (3.7) | 0.147 |
| **CVC-related** | 3 (1.4) | 2 (1.0) | 0.039 | 4 (1.1) | 1 (1.9) | 0.061 |
| **cIAI** | 23 (10.9) | 11 (5.5) | 0.199 | 25 (7.0) | 9 (16.7) | 0.303 |
| **Mucositis** | 3 (1.4) | 1 (0.5) | 0.095 | 3 (0.8) | 1 (1.9) | 0.088 |
| **ABSSTI** | 1 (0.5) | 1 (0.5) | 0.003 | 1 (0.3) | 1 (1.9) | 0.154 |
| **Biliary tract infection** | 23 (10.9) | 19 (9.5) | 0.048 | 41 (11.5) | 1 (1.9) | 0.393 |
| **Other source** | 4 (1.9) | 4 (2.0) | 0.007 | 6 (1.7) | 2 (3.7) | 0.126 |
| **Unknown source** | 42 (19.9) | 49 (24.4) | 0.108 | 76 (21.2) | 15 (27.8) | 0.153 |
| **Immunocompromised** | 39 (18.5) | 64 (31.8) | 0.312 | 90 (25.1) | 13 (24.1) | 0.025 |
| **Surgical manoeuvres** | 57 (27.0) | 52 (25.9) | 0.026 | 91 (25.4) | 18 (33.3) | 0.174 |
| **Urological manoeuvres** | 25 (13.2) | 28 (15.0) | 0.050 | 51 (15.4) | 2 (4.4) | 0.373 |
| **Biliary tract procedures** | 22 (11.6) | 16 (8.5) | 0.102 | 34 (10.2) | 4 (8.7) | 0.053 |
| **ICU admission** | 20 (9.5) | 20 (10.0) | 0.016 | 32 (8.9) | 8 (14.8) | 0.182 |
| **Neutropenia** | 9 (4.3) | 10 (5.0) | 0.034 | 15 (4.2) | 4 (7.4) | 0.138 |
| **CVC** | 46 (23.1) | 65 (34.4) | 0.251 | 96 (28.7) | 15 (27.8) | 0.021 |
| **Long-term facility stay previous 90 days** | 92 (46.7) | 111 (59.7) | 0.262 | 173 (52.3) | 30 (57.7) | 0.109 |
| **MDR isolates previous 30 days** | 18 (9.8) | 38 (22.2) | 0.344 | 49 (16.1) | 7 (14.0) | 0.058 |
| **Antibiotic use previous 30 days** | 50 (27.3) | 66 (38.2) | 0.232 | 97 (31.7) | 19 (38.0) | 0.133 |
| **Cardiovascular disease** | 43 (20.4) | 53 (26.4) | 0.142 | 77 (21.5) | 19 (35.2) | 0.307 |
| **Heart failure** | 29 (13.7) | 40 (19.9) | 0.165 | 56 (15.6) | 13 (24.1) | 0.213 |
| **Peripheral artery disease** | 30 (14.2) | 30 (14.9) | 0.020 | 52 (14.5) | 8 (14.8) | 0.008 |
| **Cerebrovascular disease** | 16 (7.6) | 11 (5.5) | 0.086 | 24 (6.7) | 3 ( 5.6) | 0.048 |
| **Dementia** | 25 (11.8) | 16 (8.0) | 0.130 | 34 (9.5) | 7 (13.0) | 0.110 |
| **COPD** | 29 (13.7) | 40 (19.9) | 0.165 | 58 (16.2) | 11 (20.4) | 0.108 |
| **Connective tissue disease** | 7 (3.3) | 5 (2.5) | 0.049 | 12 (3.4) | 0 (0.0) | 0.263 |
| **Peptic disease** | 1 (0.5) | 4 (2.0) | 0.138 | 4 (1.1) | 1 (1.9) | 0.061 |
| **Liver disease** | 22 (10.4) | 15 (7.5) | 0.104 | 34 (9.5) | 3 (5.6) | 0.150 |
| **Diabetes** | 53 (25.1) | 51 (25.4) | 0.006 | 91 (25.4) | 13 (24.1) | 0.031 |
| **Hemiplegia** | 7 (3.3) | 4 (2.0) | 0.083 | 8 (2.2) | 3 (5.6) | 0.172 |
| **Chronic kidney disease** | 36 (17.1) | 45 (22.4) | 0.134 | 67 (18.7) | 14 (25.9) | 0.174 |
| **Malignancy** | 72 (34.1) | 76 (37.8) | 0.077 | 133 (37.2) | 15 (27.8) | 0.201 |
| **Leukaemia/lymphoma** | 8 (3.8) | 18 (9.0) | 0.213 | 21 (5.9) | 5 (9.3) | 0.129 |
| **AIDS** | 0 (0) | 3 (1.5) | 0.174 | 3 (0.8) | 0 (0.0) | 0.130 |
| **Charlson index** | 6.32 (2.79) | 6.90 (2.59) | 0.213 | 6.54 (2.74) | 7.04 (2.44) | 0.193 |
| **Pitt bacteraemia score** | 0.95 (1.53) | 1.11 (1.82) | 0.093 | 0.90 (1.55) | 1.91 (2.20) | 0.532 |

^1^Mean (SD) or Frequency (%)

^2^Standardised mean difference

Figure S1. Propensity score balance and SMD after applying the propensity score

*
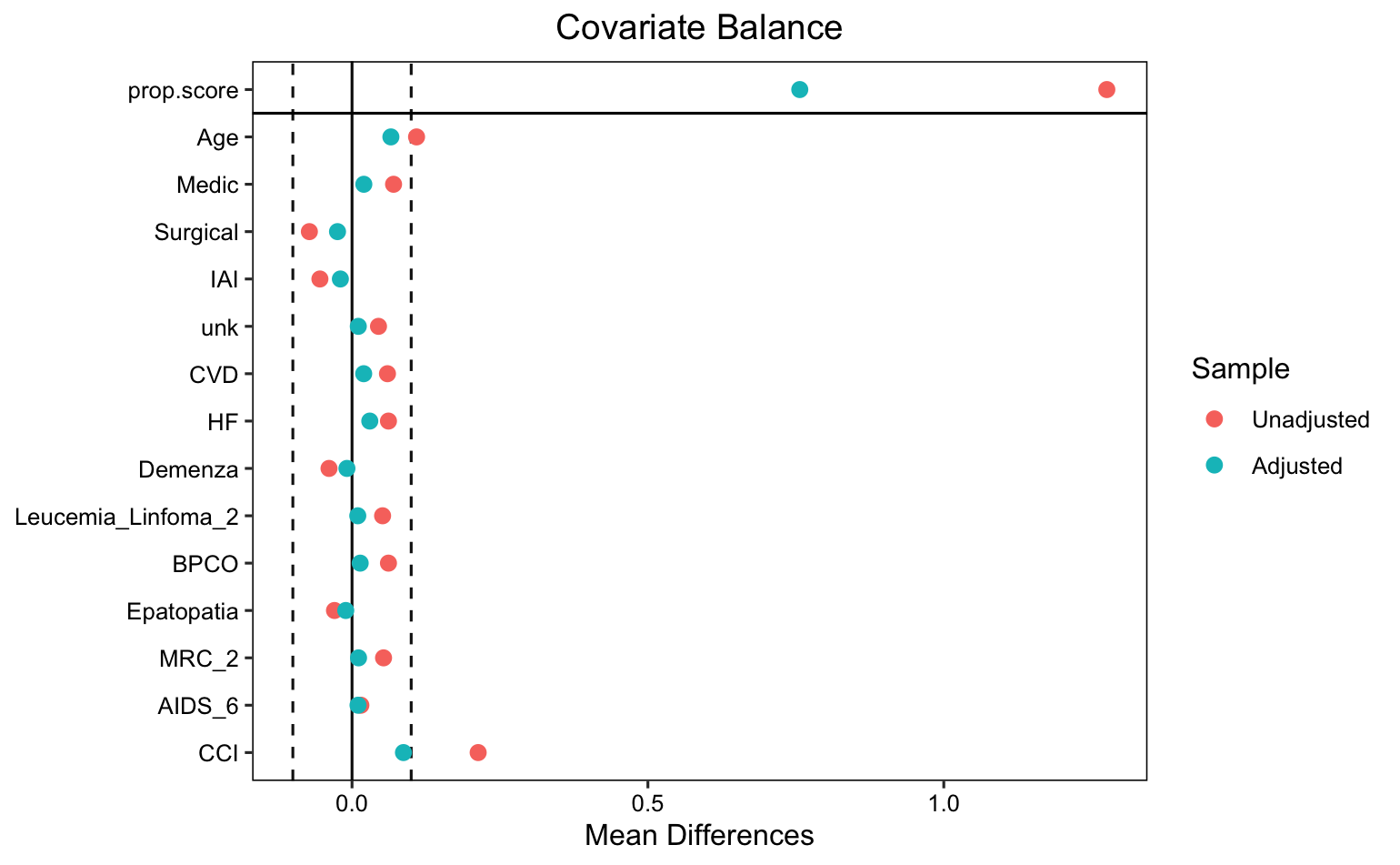
*


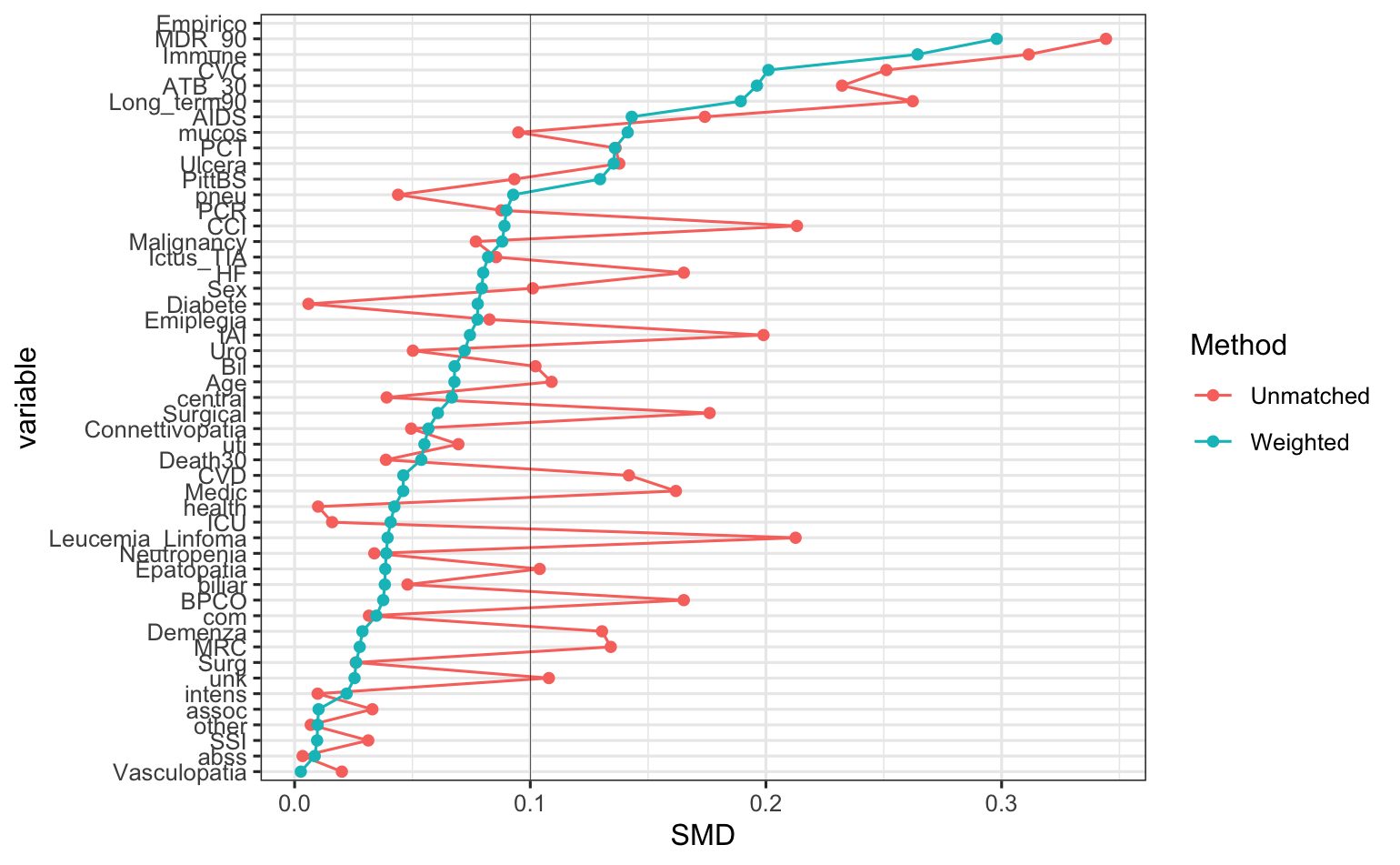


**
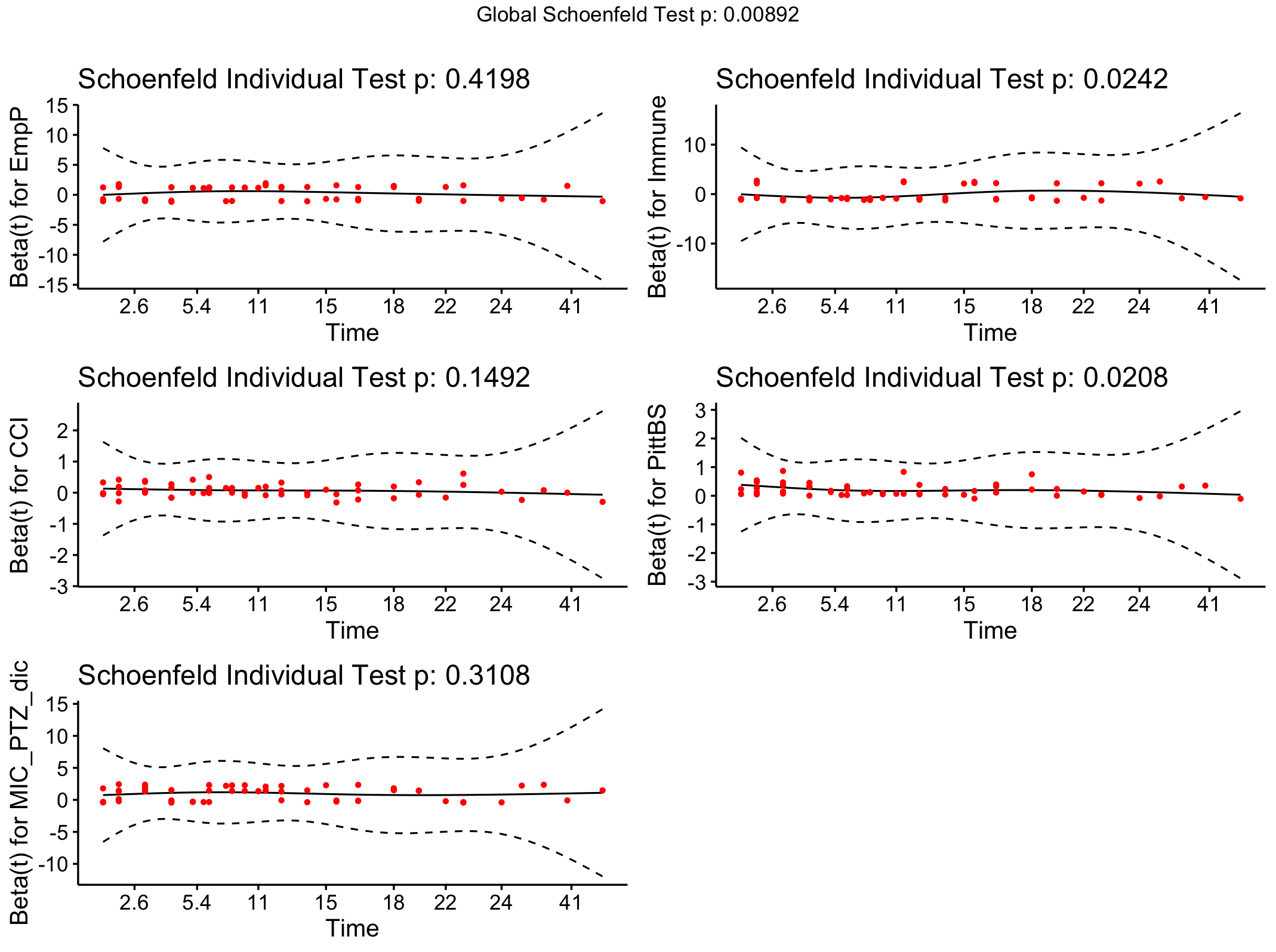
Figure S2. Cox proportional hazard assumptions verification**
